# Refractory Neuropathic Pain in the Head and Neck: Neuroanatomical and Clinical Significance of the Cervicotrigeminal Complex

**DOI:** 10.3390/life15091457

**Published:** 2025-09-17

**Authors:** Marina Raguž, Marko Tarle, Koraljka Hat, Ivan Salarić, Petar Marčinković, Ivana Bičanić, Elvira Lazić Mosler, Ivica Lukšić, Tonko Marinović, Darko Chudy

**Affiliations:** 1 Department of Neurosurgery, Dubrava University Hospital, 10000 Zagreb, Croatia; 2 School of Medicine, Catholic University of Croatia, 10000 Zagreb, Croatia; 3 Department of Maxillofacial Surgery, Dubrava University Hospital, 10000 Zagreb, Croatia; mtarle@sfzg.hr (M.T.); salaric@sfzg.unizg.hr (I.S.); luksic@kbd.hr (I.L.); 4 School of Dental Medicine, University of Zagreb, 10000 Zagreb, Croatia; 5 School of Medicine, University of Zagreb, 10000 Zagreb, Croatia; 6 Medicine of Sports and Exercise Chair, Faculty of Kinesiology, University of Zagreb, 10000 Zagreb, Croatia

**Keywords:** cervicotrigeminal complex, trigeminocervical convergence, refractory neuropathic pain, central sensitization, neuromodulation, head and neck disorders

## Abstract

Refractory neuropathic pain of the head and neck remains a major clinical challenge, particularly when mediated through the cervicotrigeminal complex (CTC), a unique anatomical hub integrating trigeminal and upper cervical nociceptive inputs. This narrative review synthesizes neuroanatomical, pathophysiological, and clinical evidence to provide a unifying framework for diagnosis and management. A structured search of PubMed, Scopus, and Web of Science identified English-language clinical and mechanistic studies addressing CTC-mediated pain, with case reports excluded unless mechanistically informative. We propose multidimensional refractoriness criteria that integrate pharmacological non-response, failed interventional strategies, and objective functional impairment. Current treatments span pharmacotherapy, peripheral interventions (nerve blocks, radiofrequency ablation), and neuromodulation at multiple network levels (occipital nerve stimulation, spinal cord stimulation, motor cortex stimulation, deep brain stimulation). Non-invasive approaches such as rTMS, tDCS, and vagus nerve stimulation are emerging but remain investigational. Advances in imaging and neurophysiological biomarkers now permit greater precision in detecting CTC dysfunction and tailoring therapy. By combining anatomical precision, mechanistic insight, and multidisciplinary strategies, this review proposes a clinically actionable definition of refractoriness and supports a stepwise, mechanism-based approach to therapy. CTC emerges as a targetable hub for diagnostic and therapeutic strategies in refractory head and neck pain.

## 1. Introduction

Neuropathic pain is defined by the International Association for the Study of Pain as pain arising as a direct consequence of a lesion or disease affecting the somatosensory nervous system [1,2]. In the head and neck region, it represents a particular diagnostic and therapeutic challenge due to the complex convergence of sensory inputs from cranial and upper cervical structures. While acute neuropathic pain may respond to standard pharmacological regimens, a considerable subset of patients progress to refractory neuropathic pain, characterized by persistent symptoms despite optimized multimodal therapy in accordance with established guidelines [3,4,5]. In this review, we define persistent neuropathic pain as pain lasting at least 3 months despite treatment with first-line agents such as tricyclic antidepressants, serotonin-norepinephrine reuptake inhibitors, or gabapentin. Epidemiological studies estimate that neuropathic pain affects approximately 6–10% of the general population [2,6,7,8]. Although head and neck neuropathic syndromes constitute a smaller fraction of these cases, they are associated with disproportionate disability, including impaired quality of life, sleep disturbance, psychiatric comorbidity, and significant socioeconomic burden through both direct healthcare costs and indirect losses such as reduced work capacity and premature retirement [8,9,10].

Central to these conditions is the cervicotrigeminal complex (CTC), a functional and anatomical hub comprising the spinal trigeminal nucleus, particularly its caudal portion, and the dorsal horns of the upper cervical spinal cord. The term CTC, sometimes also referred to as the trigeminal cervical complex, reflects this convergence and is used variably across disciplines [11]. In this review, we retain “CTC” for consistency with prior neuroanatomical literature. Together, these structures integrate nociceptive input from trigeminal and C1–C3 afferents [11,12,13,14]. This convergence provides the substrate for referred pain between cranial and cervical territories, a hallmark of clinical entities such as cervicogenic headache, occipital neuralgia, and mixed craniofacial neuropathic syndromes [15,16,17]. Experimental neurophysiology confirms that second-order neurons in the CTC respond to both trigeminal and cervical inputs, explaining overlapping pain phenotypes and the diagnostic uncertainty they often create [18,19]. While the role of the CTC has been extensively studied in migraine and trigeminal autonomic cephalalgias [20,21,22], its specific contribution to refractory neuropathic pain of the head and neck remains insufficiently characterized. Most reviews have concentrated on primary headaches or isolated trigeminal neuralgia [16,23], leaving a gap in the understanding of mixed cranio-cervical pain syndromes. Recent consensus work in headache medicine [24,25] highlights the lack of standardized definitions and treatment pathways for refractory conditions, while advances in high-resolution MR neurography and functional connectivity imaging have begun to map trigeminocervical pathways in unprecedented detail [26,27].

Against this background, the present review synthesizes anatomical, pathophysiological, diagnostic, and therapeutic evidence on the CTC in the context of refractory neuropathic pain of the head and neck. By integrating classical neuroanatomical insights with contemporary clinical and imaging data, we aim to provide a clinically actionable framework that links structure–function relationships with diagnostic strategies and stepwise treatment approaches. Rather than addressing pain syndromes in isolation, this review positions the CTC as a unifying hub and conceptual anchor for mechanism-based intervention in refractory craniofacial neuropathic pain.

## 2. Methods

This work was conducted as a structured but non-systematic narrative review. We performed a structured literature search in PubMed, Scopus, and Web of Science using combinations of the following keywords: cervicotrigeminal complex, trigeminocervical convergence, neuropathic pain, occipital neuralgia, cervicogenic headache, neuromodulation, and refractory pain. Eligible studies were restricted to English-language publications. Both clinical (human) and mechanistic (preclinical) studies were considered if they addressed diagnostic, pathophysiological, or therapeutic aspects relevant to CTC-mediated pain. Reference lists of reviews and included studies were manually screened to identify additional sources. Given the narrative design of this review, no formal inclusion/exclusion criteria were applied, and case reports were not systematically excluded.

Given the heterogeneous nature of the literature, no formal quality assessment or meta-analysis was performed. Instead, findings were synthesized to highlight converging anatomical, pathophysiological, and clinical themes relevant to refractory head and neck neuropathic pain. This synthesis was qualitative and exploratory in nature. Two authors independently screened titles/abstracts; disagreements were resolved by discussion. No formal risk-of-bias assessment was performed due to the diversity of study designs (e.g., basic science, observational, interventional) and the narrative nature of the review. However, potential methodological limitations were considered when interpreting the strength and generalizability of conclusions.

## 3. Neuroanatomy of the Cervicotrigeminal Complex

**Figure 1:**
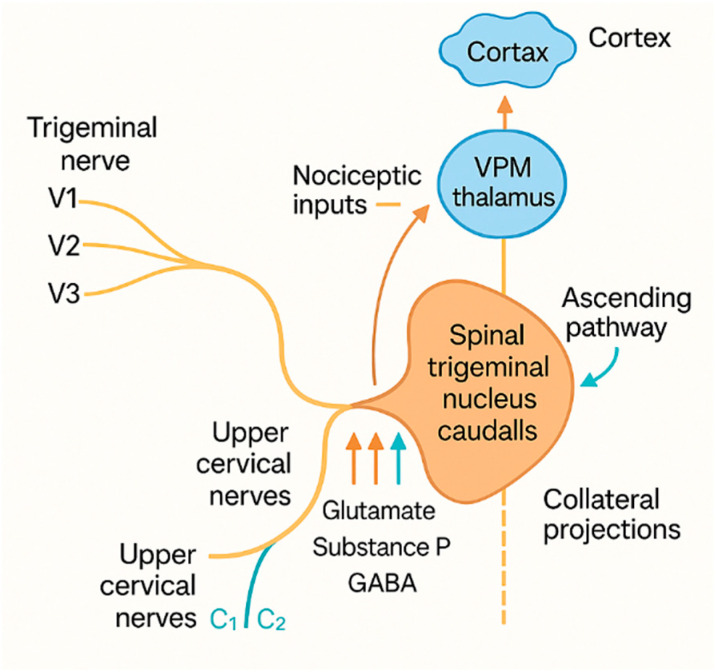
Schematic representation of the CTC. The caudal spinal trigeminal nucleus forms a continuum with the dorsal horn of the upper cervical spinal cord (C1–C3), allowing for the convergence of trigeminal (V1–V3) and cervical (C1–C3) nociceptive afferents. This integration provides the anatomical substrate for referred pain between cranial and cervical regions. Excitatory neurotransmitters (e.g., glutamate, substance P) and inhibitory mediators (e.g., GABA) modulate signal transmission within the CTC. These outputs influence both ascending nociceptive pathways and collateral projections involved in autonomic features. Abbreviations: CTC, cervicotrigeminal complex; V1–V3, trigeminal divisions; C1–C3, upper cervical nerves; PAG, periaqueductal gray; CGRP, calcitonin gene-related peptide; GABA, γ-aminobutyric acid.

The CTC is not a passive relay station but a dynamic integrative hub whose connectivity underpins both the clinical manifestations and therapeutic targeting of neuropathic pain in the head and neck. Anatomically, the CTC is centered on the spinal trigeminal nucleus, particularly its caudal portion, the nucleus caudalis, which is continuous caudally with the dorsal horn of the upper cervical spinal cord [11,28]. This structural continuum establishes direct anatomical and physiological links between trigeminal sensory pathways and cervical somatosensory inputs, enabling convergence of nociceptive signals from distinct territories [13,29]. Primary afferents from the trigeminal nerve descend within the spinal trigeminal tract before terminating in the nucleus caudalis, while afferents from the upper cervical nerves (C1–C3) enter the dorsal horn of the cervical cord and converge via interneuronal networks onto shared second-order neurons within the CTC [19,30]. This convergence provides the neurobiological substrate for referred pain between cranial and cervical regions, explaining how cervical pathology may present as craniofacial pain and vice versa [15,16] (Figure 1).

The caudal trigeminal nucleus exhibits a clear somatotopic organization: the perioral region is represented rostrally, while more peripheral facial territories are mapped caudally [31,32]. Neuroimaging studies using diffusion tensor imaging (DTI) and functional MRI support these projection patterns in vivo, confirming C1–C3 convergence onto neurons processing ophthalmic division (V1) input [26]. This overlap explains the spread of pain from occipital to orbital or frontal regions, a hallmark of cervicogenic headache and occipital neuralgia [16,17]. From the CTC, second-order neurons ascend via the trigeminothalamic tract to the ventroposteromedial (VPM) thalamus and associated relay structures, ultimately projecting to the primary (S1) and secondary (S2) somatosensory cortices [33]. Collateral projections to the periaqueductal gray (PAG) and parabrachial nucleus provide a structural basis for the autonomic features frequently observed in CTC-mediated syndromes, such as lacrimation, conjunctival injection, and nasal congestion [16,34]. At the neurochemical level, synaptic activity in the pars caudalis is mediated by excitatory transmitters such as glutamate and substance P, counterbalanced by inhibitory mediators including GABA and glycine. Calcitonin gene-related peptide (CGRP) has also emerged as a critical neuropeptide linking the CTC to migraine and refractory neuropathic pain [35,36]. This precise anatomical convergence explains the complex referral patterns encountered clinically and provides the rationale for targeted interventions, including greater occipital nerve blocks, cervical medial branch blocks, radiofrequency ablation, occipital nerve stimulation (ONS), and high cervical spinal cord or deep brain stimulation (SCS/DBS), all of which act by modulating nociceptive processing within the CTC [37,38]. A thorough understanding of this network is fundamental for the development and selection of effective strategies to manage refractory neuropathic pain in the head and neck.

## 4. Pathophysiology of CTC-Mediated Pain

The pathophysiology of CTC-mediated pain reflects an interplay of molecular, cellular, and systems-level mechanisms that sustain chronic pain states in the head and neck. Anatomical overlap of trigeminal and upper cervical afferents onto common second-order neurons in the spinal trigeminal nucleus caudalis provides the substrate for referred pain and overlapping symptoms across cranial and cervical territories [13,16]. This arrangement permits convergent facilitation, whereby persistent nociceptive activity in one region amplifies input from another, a process that clinically explains how occipital neuralgia may present with orbital or frontal pain, and conversely, trigeminal neuropathy with occipital features [17].

A central hallmark of chronic CTC-mediated pain is central sensitization, characterized by heightened excitability of neurons in the nucleus caudalis and upper cervical dorsal horn following persistent peripheral input. Key mechanisms include excessive glutamatergic transmission and NMDA receptor activation [39], receptor phosphorylation and AMPA receptor trafficking [40], reduced GABAergic and glycinergic inhibition [19], and the recruitment of wide dynamic range neurons with enlarged receptive fields. Clinically, these processes manifest as hyperalgesia, allodynia, and spatial expansion of pain. Animal models confirm that repetitive dural or cervical stimulation can induce prolonged hyperexcitability within CTC [37].

**Figure 2:**
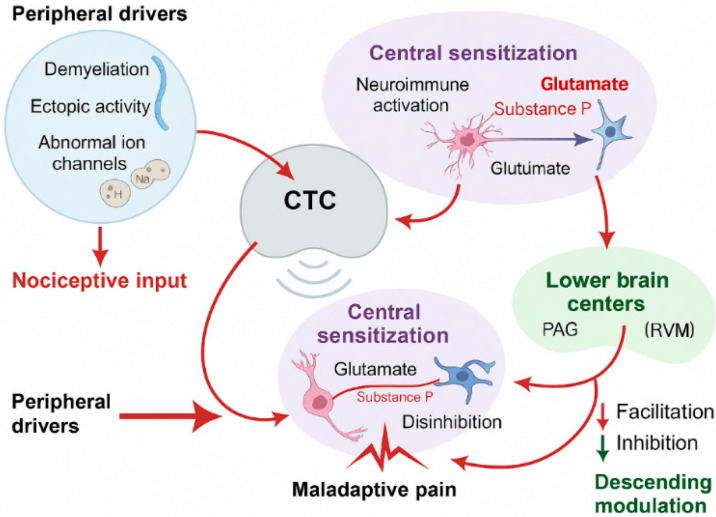
Peripheral drivers enhance nociceptive input to the CTC. Persistent input leads to central sensitization, characterized by the release of excitatory neurotransmitters (e.g., glutamate, substance P), disinhibition of pain pathways, and neuroimmune activation. Descending modulation from brainstem centers (PAG and RVM) normally balances excitatory and inhibitory influences. In refractory pain states, inhibitory control is reduced, and facilitation predominates, sustaining maladaptive pain signaling. Abbreviations: CTC, cervicotrigeminal complex; PAG, periaqueductal gray; RVM, rostral ventromedial medulla.

Sustained afferent drive further activates glial networks, with microglia and astrocytes releasing IL-1β, TNF-α, chemokines, and brain-derived neurotrophic factor (BDNF) [41,42]. BDNF downregulates KCC2, reducing inhibitory tone and promoting depolarizing GABA responses, thereby reinforcing maladaptive plasticity. Peripheral factors such as trauma, compression, post-viral neuritis, demyelination, or ion channel dysregulation may perpetuate these central changes, with NaV1.7, NaV1.8, and CaV2.2 channel upregulation driving ectopic firing in injured cranial and cervical nerves [43]. Under normal circumstances, descending control from the PAG and rostral ventromedial medulla (RVM) balances inhibition and facilitation. In refractory states, however, descending facilitation predominates through serotonergic and glutamatergic pathways, while noradrenergic and GABAergic inhibition diminishes [44]. This imbalance not only maintains pain but also links nociceptive processing with affective and cognitive domains, consistent with the high prevalence of depression, anxiety, and sleep disturbance in CTC-mediated syndromes [45]. The interaction of peripheral drive, central sensitization, neuroinflammation, and impaired descending inhibition establishes a self-perpetuating feedback loop that resists conventional pharmacotherapy (Figure 2).

Since no single mechanism explains refractoriness, therapeutic strategies should target multiple nodes simultaneously: reducing peripheral input (nerve blocks, decompression, ion channel modulators), suppressing central hyperexcitability (gabapentinoids, NMDA antagonists, GABAergic enhancers), attenuating glial activation (cytokine or microglial inhibitors), and restoring descending inhibition (SNRIs, neuromodulation of PAG–RVM circuits).

## 5. Clinical Presentation

Neuropathic and mixed pain syndromes of the head and neck display marked heterogeneity, reflecting the complex convergence within the CTC and resulting in overlapping phenotypes that often elude strict diagnostic categorization. Shared receptive fields of trigeminal and upper cervical afferents enable referred pain patterns that obscure the primary site of pathology, leading to diagnostic delays and complicating management [13,17]. Occipital neuralgia is among the most recognizable CTC-mediated syndromes, presenting with paroxysmal stabbing pain in the occipital distribution that may radiate to orbital or frontal regions, frequently mimicking migraine or trigeminal autonomic cephalalgias [15,16]. Cervicogenic headache similarly originates from upper cervical pathology with referral into trigeminal territories, and is typically aggravated by neck movement or posture. Diagnostic certainty requires adherence to ICHD-3 criteria, integrating both clinical findings and radiological correlation [11,21].

Other neuralgic syndromes, including glossopharyngeal neuralgia, Eagle syndrome, and post-herpetic neuralgia, may overlap with CTC-mediated presentations. Post-traumatic trigeminal neuropathy and persistent idiopathic facial pain illustrate how central sensitization can broaden the pain field into cervical regions [23,46,47]. Beyond neuralgic disorders, musculoskeletal, dental, otorhinolaryngological, and myofascial conditions also converge on the CTC. Temporomandibular disorders, myofascial trigger points, atypical odontalgia, sinus disease, and referred otalgia may all mimic or exacerbate neuropathic pain phenotypes [48,49,50,51]. In addition, autonomic features such as lacrimation, conjunctival injection, ptosis, and nasal congestion, as well as sensory disturbances including allodynia, dysesthesia, and hypoesthesia, are frequently encountered in CTC-mediated pain. These reflect collateral projections from the caudal trigeminal nucleus to brainstem autonomic centers and maladaptive central neuroplasticity [19,34]. Such manifestations blur clinical boundaries with trigeminal autonomic cephalalgias, migraine, and other primary headache disorders [25,30].

Given this wide spectrum, recognition of overlapping phenotypes and their integration into structured diagnostic pathways is essential. Table 1 provides a comparative overview of key clinical features, diagnostic clues, and relevant specialties across neuropathic, musculoskeletal, dental, and otorhinolaryngological entities converging on the CTC.

## 6. Diagnostic and Therapeutic Framework for Refractory Neuropathic Pain in the Head and Neck

Neuropathic pain of the head and neck mediated by the CTC presents a distinct diagnostic challenge due to the anatomical convergence of trigeminal and upper cervical afferents onto shared second-order neurons within the spinal trigeminal nucleus caudalis. This arrangement enables bidirectional nociceptive referral between cranial and cervical territories, generating overlapping and sometimes misleading symptom patterns that obscure the primary pain generator. Such convergence carries a high risk of misclassification and highlights the importance of an anatomically informed, multidisciplinary approach, with the designation “refractory” reserved for cases that have undergone comprehensive evaluation and optimal treatment [13,17].

The diagnostic process begins with a detailed history and focused neurological examination, addressing pain onset, temporal pattern, quality, distribution, and associated autonomic or neurological features [21]. Neuropathic descriptors such as burning, electric shock-like, or stabbing pain often suggest central sensitization mechanisms [2]. Sensory mapping across trigeminal and upper cervical dermatomes is valuable to identify hypoesthesia, hyperalgesia, or mechanical allodynia spanning both regions, consistent with convergent processing in the CTC [19]. Cranial nerve testing, cervical range of motion, and palpation of occipital nerve emergence points provide further localization clues, while reproduction of pain with neck movement or posture supports a cervicogenic contribution. Red flags, including progressive neurological deficit, constitutional symptoms, new-onset pain in older age, or risk factors for malignancy or infection, mandate early MRI of the brain, craniocervical junction, and cervical spine. Structural exclusion is essential: MRI is first-line to identify demyelination, Chiari malformation, syringomyelia, cervical spondylosis, vascular malformations, or neoplasms at the craniocervical junction [72]. In suspected peripheral nerve injury, high-resolution MR neurography and DTI can detect microstructural changes [26,73]. Neurophysiological investigations, including trigeminal somatosensory evoked potentials (TSEPs), blink reflex testing, and quantitative sensory evaluation, further assess brainstem pathway function and nociceptive processing. Targeted diagnostic nerve blocks (greater occipital, supraorbital, auriculotemporal) serve both diagnostic and prognostic purposes, identifying peripheral nociceptive drivers and predicting response to interventional therapies [74].

At present, there is no universally accepted definition of refractoriness in neuropathic pain of the head and neck, particularly in disorders mediated by CTC. Studies apply heterogeneous criteria, with some focusing solely on pharmacological failure and others neglecting functional or anatomical considerations. To address this, we propose a pragmatic, multidisciplinary framework adapted to the specific neuroanatomical and clinical features of CTC-related pain (Table 2). In this context, refractoriness should be defined as persistent moderate-to-severe pain (NRS ≥ 4/10) lasting ≥3 months despite optimized multimodal therapy, documented failure of at least two first-line pharmacologic classes (gabapentinoids, tricyclic antidepressants, or serotonin–norepinephrine reuptake inhibitors), each administered at maximally tolerated doses for ≥6 weeks [3,5], lack of sustained benefit from at least one anatomically targeted interventional procedure (e.g., occipital nerve block or pulsed radiofrequency), indicating peripheral resistance [75], objective evidence of functional impairment or reduced quality of life using validated tools such as the Brief Pain Inventory (BPI), Headache Impact Test (HIT-6), or DN4 questionnaire [76], and exclusion of surgically remediable or secondary causes such as neoplasms, Chiari malformation, or demyelination [17]. Table 2 summarizes these proposed refractoriness criteria and may serve as a foundation for clinical consensus and future study design.

**Figure 3:**
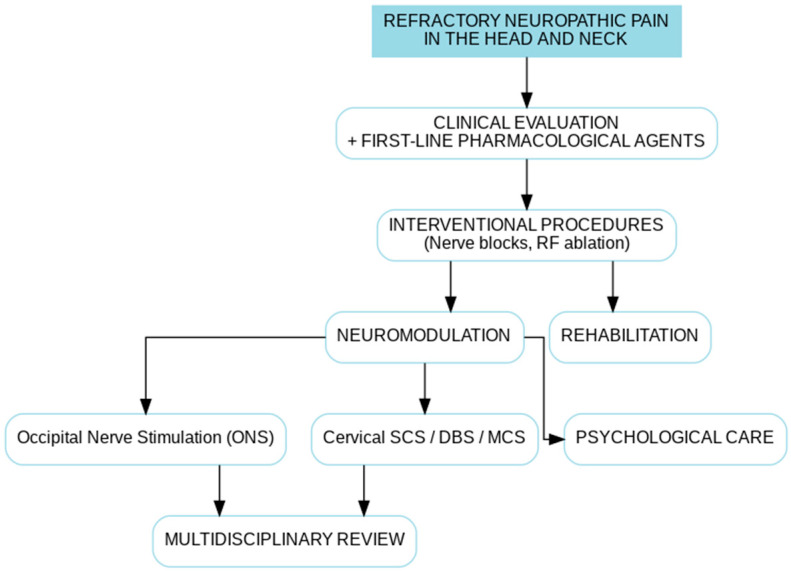
Clinical decision algorithm and multimodal management pathway for refractory neuropathic pain of the head and neck. The framework illustrates a stepwise escalation from first-line pharmacological therapies to targeted interventional procedures, invasive and non-invasive neuromodulation, and comprehensive rehabilitative and psychological strategies. The model emphasizes integration rather than sequential isolation, with multidisciplinary input at each stage to address peripheral drivers, central sensitization, and network-level dysfunction. The algorithm incorporates refractoriness criteria (Table 2) and highlights the predictive value of diagnostic nerve blocks in guiding escalation to neuromodulation.

Management of refractory neuropathic pain in the head and neck requires a tiered and multimodal strategy, tailored to both underlying mechanisms and symptom severity. Treatment typically progresses from conservative pharmacological regimens to invasive neuromodulation, with escalation considered only after less invasive approaches fail. A stepwise management algorithm is summarized in Figure 3.

Pharmacological therapies, including gabapentinoids, tricyclic antidepressants, serotonin–norepinephrine reuptake inhibitors, and topical agents, remain first-line options. Their efficacy is supported by randomized controlled trials (RCTs) and meta-analyses in general neuropathic pain populations; however, effectiveness in refractory head and neck pain is often modest, and systemic adverse effects may limit long-term use [3,5].

When pharmacological measures are insufficient, interventional procedures such as occipital and trigeminal nerve blocks may provide both diagnostic and short-term therapeutic benefit. Although generally safe and repeatable, their effect is usually transient [75,77]. Pulsed radiofrequency (PRF) of peripheral nerves or cervical dorsal root ganglia has also been explored, with heterogeneous results across small studies [53]. Peripheral neuromodulation with ONS has shown promise, particularly in occipital neuralgia and overlapping trigeminocervical pain syndromes. Multiple RCTs in chronic migraine and observational studies in occipital neuralgia suggest benefit, though complications such as lead migration and infection remain limitations [78,79]. SCS, especially with high-frequency or burst paradigms, has been applied to upper cervical and trigeminocervical pain syndromes with encouraging but preliminary results [80,81]. Motor cortex stimulation (MCS) has a more established role in refractory trigeminal neuropathic pain and central post-stroke pain, with functional imaging suggesting restoration of disrupted CTC connectivity. Clinical benefit has been reported in 40–60% of patients, though risks such as seizure induction and variability in long-term efficacy remain [82,83,84,85]. At the far end of the therapeutic spectrum, DBS remains an experimental approach, reserved for highly refractory cases. Proposed targets include the PAG, posterior hypothalamus, ventral posteromedial thalamus, and anterior cingulate cortex, reflecting the multidimensional nature of pain processing. While case series report potential benefit, heterogeneity of targets, surgical risks, and ethical considerations currently preclude standardized use [86,87].

Table 3 provides a comparative overview of these therapeutic strategies, highlighting mechanisms of action, limitations, and the general strength of supporting evidence.

Among neuromodulatory strategies, non-invasive approaches warrant particular attention because of their favorable safety profile and ease of application (Table 4). Techniques such as repetitive transcranial magnetic stimulation (rTMS), transcranial direct current stimulation (tDCS), non-invasive vagus nerve stimulation (nVNS), and transcutaneous electrical nerve stimulation (TENS) offer additional therapeutic options with relatively low risk (Table 4). While the strongest evidence derives from studies in primary headache disorders, early data indicate potential benefit in neuropathic pain syndromes. However, protocols remain heterogeneous, and further randomized controlled trials are needed to define efficacy, optimal stimulation parameters, and patient selection [88,89].

At every stage, rehabilitation plays a critical supportive role. Cervical mobility training, postural correction, and physiotherapy help reduce musculoskeletal contributors to nociceptive load. Psychological interventions, particularly cognitive–behavioral therapy, address mood and anxiety comorbidities that exacerbate pain via shared neural circuits. Sleep optimization is equally important, given the detrimental effects of sleep disruption on descending inhibitory pathways [95]. Adjunctive measures, including orofacial rehabilitation as well as targeted dental and ENT interventions, remain indispensable to ensure comprehensive, multidisciplinary care [23,90].

## 7. Discussion

CTC occupies a distinctive position in pain neurobiology. Rather than a passive relay, it functions as a dynamic hub where trigeminal and upper cervical afferents converge onto shared second-order neurons [11,13]. This anatomical continuity between the spinal trigeminal nucleus caudalis and the dorsal horn of C1–C3 [30] creates a substrate for bidirectional cross-talk. Clinically, this explains why cervical pathology can mimic craniofacial neuralgia, and conversely, why craniofacial pathology may radiate to the occiput. As a result, diagnostic boundaries often remain blurred, and misclassification or delayed treatment is common [15,16,18].

Despite decades of research, the pathophysiology of CTC-mediated refractory pain remains incompletely characterized. Many studies describe these syndromes under broad labels such as “atypical” or “mixed” pain, which risks obscuring their specific neurobiological underpinnings. Experimental evidence indicates that sustained afferent input can induce central sensitization within the caudalis, sustained by NMDA receptor phosphorylation, disinhibition of inhibitory networks, and glial-driven neuroinflammation [19,39,41]. Recent preclinical data further suggest that sex-specific microglial subtypes may contribute differentially to pain maintenance, highlighting future avenues for targeted therapies [96,97]. Once established, this maladaptive state expands receptive fields, amplifies nociceptive gain, and transforms localized injury into widespread, treatment-resistant pain. Descending facilitation from the PAG–RVM system, often coupled with mood and sleep dysregulation, may further perpetuate chronicity [95]. In addition, chronic postoperative pain following cervicofacial cancer surgeries, such as neck dissection, mandibulectomy, or parotidectomy, may involve the CTC due to peripheral nerve injury and central sensitization. Further research is needed to better characterize CTC-mediated mechanisms in this important patient population. Similar pathophysiological mechanisms, namely, central sensitization, impaired inhibition, and descending facilitation, are also observed in other refractory pain syndromes such as brachial plexus avulsion, post-thoracotomy pain, and spinal root injury. These parallels underscore that CTC-mediated pain shares common neuroplastic pathways with neuropathic conditions in anatomically distant regions. Taken together, these findings suggest that CTC-mediated refractory pain represents not merely “neuropathic pain in another location,” but a network disorder with system-level dysfunction.

Therapeutic approaches should therefore reflect this complexity. As outlined in Table 2, definitions of refractoriness need to integrate duration, pharmacological and interventional non-response, functional impact, and exclusion of surgically remediable causes. Conventional pharmacology remains the starting point, but often provides limited relief. Gabapentinoids, antidepressants, and topical agents are supported by trials in generalized neuropathic pain [3,5], yet their effectiveness in CTC syndromes is modest, and systemic side effects can restrict prolonged use. Interventional strategies such as nerve blocks or pulsed radiofrequency retain diagnostic value, but therapeutic effects are typically transient [74,75]. Neuromodulation, summarized in Table 3 and Figure 3, provides a rational escalation, as it directly targets the network circuits implicated in refractoriness. ONS may modulate caudalis activity [78,79]. High cervical SCS has shown promise in restoring inhibitory connectivity [80,81]. MCS has demonstrated benefit in refractory trigeminal neuropathic pain and central post-stroke pain, potentially by recalibrating thalamo-cortical and brainstem nociceptive circuits [82,83,84,85]. At the furthest end of the interventional spectrum, DBS remains experimental, with heterogeneous targets explored in small series [86,87].

Recent studies have explored DBS targeting regions such as the PAG, anterior cingulate cortex, and ventral posteromedial thalamus, with varying degrees of success [84,85,98,99]. Although results remain preliminary, DBS may offer relief in select patients with refractory neuropathic pain when conventional and peripheral interventions have failed. Direct nerve stimulation, particularly of the occipital and auriculotemporal nerves, is also gaining interest as a more targeted and less invasive alternative [100,101]. Ongoing trials aim to define optimal parameters and patient selection criteria for these approaches. Future directions may include biomarker-informed neurosurgical planning and closed-loop stimulation systems tailored to individual pain signatures [77,87]. Nevertheless, even advanced neuromodulatory strategies are not universally effective. By the time many patients are labeled “refractory,” mechanisms such as neuroinflammation and descending facilitation may already be entrenched. This underscores the importance of integrated, layered interventions applied earlier in the course of disease, rather than sequential monotherapies. Non-invasive neuromodulation techniques, including rTMS, tDCS, nVNS, and TENS (Table 4), are of particular interest, given their safety and ability to modulate pain networks without surgical risk [88,91,92,93,94]. A structured treatment algorithm is provided in Figure 3, outlining escalation from pharmacologic management to advanced neuromodulation based on refractoriness criteria and diagnostic response.

Future directions may involve bridging molecular insights with clinical translation. Potential avenues include microglial and astrocytic modulators (e.g., P2 × 4, CCL4/CCR5 axis), novel NMDA antagonists, anti-inflammatory biologics, and regenerative cell-based approaches [42,102]. In parallel, advanced neuroimaging and connectivity-based biomarkers (e.g., MR neurography, DTI, AI-driven pattern recognition) could refine patient stratification and treatment personalization [103].

Importantly, the current evidence base remains limited. Most studies are small, heterogeneous, and often extrapolated from headache or generalized neuropathic pain cohorts. Definitions of refractoriness lack standardization, and outcome measures are inconsistent, limiting comparability across trials. Moreover, the variability in study design and quality among included sources may limit the generalizability of conclusions and underscores the need for prospective, high-quality research in this area. Collaborative, multicenter efforts will be essential to strengthen the field. By integrating anatomy, mechanism, and therapy, this review highlights CTC-mediated refractory pain as a definable clinical entity and a potential target for mechanism-informed interventions. A shift toward multidisciplinary, network-level approaches may help move the field beyond descriptive syndromes and toward evidence-based, patient-centered care. Where direct studies on cervicotrigeminal pain are lacking, extrapolations from migraine or generalized neuropathic pain literature are noted as such and should be interpreted with caution.

## 8. Conclusions

Refractory neuropathic pain of the head and neck is increasingly recognized not as a collection of atypical syndromes, but as a disorder with a definable neurobiological basis in CTC dysfunction. This review positions the CTC as a useful framework for understanding trigeminocervical convergence, central sensitization, and treatment resistance, and for moving beyond purely symptom-based classifications toward mechanism-informed diagnosis. By integrating neuroanatomical insights with structured definitions of refractoriness and tiered therapeutic strategies, we suggest that clinical care should adopt multidimensional criteria that encompass duration, pharmacological and interventional response, functional impact, and exclusion of surgically remediable causes. Based on current evidence, we recommend a layered therapeutic approach tailored to the pathophysiological mechanisms involved, starting with pharmacotherapy, progressing through targeted interventions (e.g., nerve blocks, PRF), and advancing to neuromodulation when appropriate. Progress in management will likely depend on earlier, layered interventions that address peripheral drivers, central sensitization, and maladaptive network activity in parallel. Such approaches, combining pharmacology, interventional techniques, and neuromodulation within a coordinated multidisciplinary framework, may improve outcomes, particularly when supported by advanced imaging and biomarker-guided patient selection. Clinicians should also consider CTC dysfunction in cases of post-surgical craniofacial pain and other refractory neuropathic syndromes with overlapping mechanisms.

Looking ahead, consensus definitions, high-quality multicenter trials, and translational innovation directed at neuroimmune and glial signaling will be essential. By linking anatomical understanding with mechanistic insights and technological advances, there is an opportunity to reframe CTC-mediated pain as a better characterized and more manageable condition, while acknowledging that further evidence is needed before it can be fully predicted, controlled, or prevented.

## Figures and Tables

**Table 1:** Overview of neuropathic, musculoskeletal, maxillofacial, dental, and otorhinolaryngological conditions converging on the CTC, with overlapping features and diagnostic clues.

Disorder	Pain Duration	Pain Distribution	Triggers	Diagnostic Clues	Primary Innervation	Pain Type	Typical Diagnostic Tests	Key References
Cervicogenic headache	Chronic, recurrent	Unilateral occipital → fronto-orbital	Neck movement, sustained posture	Reduced cervical ROM, improvement after diagnostic block	C2–C3 dorsal roots, trigeminocervical convergence	Deep, dull, non-pulsatile	Cervical imaging, diagnostic block	[11,52]
Occipital neuralgia	Paroxysmal, seconds–minutes	Greater/lesser occipital nerves → orbit, temple	Palpation, head movement	Tenderness over nerve exit, pain relief with block	Greater/lesser occipital nerves (C2)	Sharp, stabbing	Diagnostic nerve block	[53,54]
Temporomandibular disorders (TMD)	Subacute–chronic	Jaw, face, ear	Mouth opening, clenching	Joint clicks, tenderness of masseter/TMJ	V3 (mandibular branch)	Myofascial, mixed	MRI TMJ, dental exam	[48,55]
Pulpitis (acute, irreversible)	Acute	Diffuse facial pain, jaws, temporal region, ear	Thermal or mechanical stimuli	Pulp vitality tests, Percussion tests	V2–V3	Sharp, intense, spontaneous, lingering	dental examination	[56]
Periapical periodontitis	Dull, poorly localized	Face, neck and ear of the affected side	Mechanical stimuli	The tooth feels high or extruded in occlusion, and pain increases in occlusal contact	V2–V3	Deep, dull, continuous aching	dental examination, Panoramic X-ray, CBCT	[56]
Post-extraction alveolitis (dry socket)	Persistent	Ear, temporal region, the affected side of the neck	Mechanical stimuli, chewing	Empty tooth alveolus, exposed bone, foul odor or taste	V2–V3	Severe, throbbing, deep	Dental examination	[57]
Iatrogenic dental injury (following endodontic treatment, implant placement or tooth extraction)	Persistent	Adjacent teeth, ipsilateral jaw, temporal region, ear, neck	Mechanical or chemical stimuli	Pain after dental treatment; examination is usually unremarkable	V2–V3	Dysesthesia	dental examination, Panoramic X-ray, CBCT	[56]
Atypical odontalgia	Persistent	Maxillary/mandibular teeth, diffuse face	Chewing, dental procedures	Pain without an odontogenic cause	V2–V3	Burning, aching, dysesthesia	Panoramic X-ray, dental examination, CBCT	[50,58]
Sinusitis-related facial pain	Acute or chronic	Maxillary, frontal, periorbital	Positional, nasal congestion	Nasal discharge, sinus tenderness	V1–V2	Pressure-like	CT sinuses	[59,60]
Glossopharyngeal neuralgia	Paroxysmal, seconds	Throat, base of tongue, ear	Swallowing, talking	Trigger points in the tonsillar fossa	CN IX	Electric shock-like	MRI, neuro exam	[61,62]
Eagle syndrome	Chronic, intermittent	Throat, jaw, ear	Swallowing, head rotation	Palpable styloid process, CT elongation	CN V, VII, IX, X	Mixed neuropathic	CT 3D reconstruction	[63,64]
Post-herpetic neuralgia (PHN)	Chronic > 3 months	Ophthalmic division (V1), sometimes C2–C3	Spontaneous, tactile	Allodynia, history of shingles	V1 ± cervical DRG	Burning, neuropathic	Clinical, dermatomal mapping	[65,66]
Migraine with occipital radiation	Hours–days, recurrent	Hemicranial → occipital/neck	Stress, sleep, triggers	Photophobia, aura, relief with triptans	Trigeminovascular system, C2 afferents	Pulsatile, throbbing	Clinical (ICHD-3 criteria)	[20,67]
Chronic otalgia (non-otologic)	Persistent	Ear, periauricular, pharyngeal	Swallowing, chewing	Normal otoscopy	CN V, VII, IX, X	Referred pain	ENT exam, exclude malignancy	[68,69]
Myofascial pain (SCM, trapezius)	Chronic	Neck, jaw, face	Palpation, posture	Trigger points	Cervical muscle nociceptors → CTC	Dull, aching, referred	Palpation, EMG if needed	[70,71]
Persistent idiopathic facial pain (PIFP)	Continuous, daily	V2–V3, diffuse face	Nonclear	No identifiable pathology	CN V (central)	Burning, aching	Diagnosis of exclusion	[23,47]

Abbreviations: ON, occipital neuralgia; CH, cervicogenic headache; GN, glossopharyngeal neuralgia; PHN, post-herpetic neuralgia; PIFP, persistent idiopathic facial pain; PTN, post-traumatic trigeminal neuropathy; TMD, temporomandibular disorder; AO, atypical odontalgia; ENT, ear, nose, and throat.

**Table 2:** Proposed refractoriness criteria for CTC-mediated head and neck neuropathic pain.

Domain	Criterion
Duration and intensity	Persistent pain ≥ 3 months with NRS ≥ 4/10
Pharmacological failure	Non-response or intolerance to ≥2 first-line drug classes (gabapentinoids, tricyclic antidepressants, SNRIs) at maximally tolerated doses for ≥6 weeks each
Interventional failure	Lack of sustained benefit from ≥1 anatomically targeted procedure (e.g., occipital or trigeminal branch block ± pulsed radiofrequency) aligned with the pain generator
Functional impact & exclusion	Objective impairment on validated scales (e.g., BPI, HIT-6, DN4) and exclusion of surgically remediable or secondary causes (tumor, Chiari malformation, demyelination)

**Table 3:** Integrated therapeutic approaches for refractory neuropathic pain of the head and neck mediated by the CTC.

Therapeutic Strategy	Mechanism of Action	Level of Evidence (OCEBM)	Limitations	Ref.
Pharmacological therapies	Gabapentinoids: α2δ calcium channel inhibition; TCAs/SNRIs: descending inhibitory facilitation; Topical agents: sodium channel blockade	Level 1 (systematic reviews, RCTs in general neuropathic pain; limited head–neck data)	Systemic side effects, limited efficacy in refractory CTC syndromes	[3,5]
Diagnostic/therapeutic nerve blocks	Peripheral afferent interruption; diagnostic confirmation of pain generator	Level 2–3 (small RCTs, observational studies)	Transient benefit; requires repetition	[74,75]
Pulsed radiofrequency (PRF)	Neuromodulation of peripheral nerves/cervical DRG without neurodestruction	Level 3–4 (case series, small trials)	Heterogeneous protocols, variable durability	[74]
Occipital nerve stimulation (ONS)	Modulation of trigeminocervical complex activity; normalization of pain networks	Level 1–2 (RCTs in migraine, observational studies in ON)	Invasive, lead migration, infection	[78,79]
Spinal cord stimulation (SCS)	Inhibition of dorsal horn hyperexcitability and CTC input integration	Level 2–3 (case series, pilot RCTs)	Invasive; costly; limited head/neck data	[80,81]
Motor cortex stimulation (MCS)	Modulation of thalamo-cortical and brainstem nociceptive circuits	Level 2–3 (systematic reviews, observational studies)	Variable long-term efficacy; seizure risk	[82,83,84,85]
Deep brain stimulation (DBS)	Target-specific modulation (PAG, posterior hypothalamus, VPM thalamus, ACC)	Level 4 (case series; experimental)	Surgical risk; heterogeneous targets; ethical concerns	[86,87]
Non-invasive neuromodulation (rTMS, tDCS, nVNS, TENS)	Modulation of cortical and brainstem excitability; rebalancing descending pathways	Level 2–3 (RCTs in migraine/TTH; emerging in neuropathic pain)	Short-term benefit; protocol standardization needed	[88,89]
Multidisciplinary rehabilitation	Reduction in musculoskeletal nociceptive load; coping; sleep regulation	Level 2–3 (guideline-based, pragmatic trials)	Supportive rather than curative	[23,90]

Abbreviations: TCA, tricyclic antidepressant; SNRI, serotonin–norepinephrine reuptake inhibitor; DRG, dorsal root ganglion; ONS, occipital nerve stimulation; SCS, spinal cord stimulation; MCS, motor cortex stimulation; DBS, deep brain stimulation; PAG, periaqueductal gray; VPM, ventral posteromedial nucleus; ACC, anterior cingulate cortex; rTMS, repetitive transcranial magnetic stimulation; tDCS, transcranial direct current stimulation; nVNS, non-invasive vagus nerve stimulation; TENS, transcutaneous electrical nerve stimulation. Level of evidence: Classified according to the Oxford Centre for Evidence-Based Medicine, where Level 1 = systematic review/RCT, Level 2 = RCT or strong observational, Level 3 = cohort/case–control, Level 4 = case series/poor-quality studies, Level 5 = expert opinion.

**Table 4:** Non-invasive neuromodulatory approaches for refractory neuropathic pain of the head and neck (CTC-related syndromes).

Modality	Mechanism of Action	Targeted Structures	Level of Evidence (OCEBM)	Limitations	Refs.
rTMS	Modulates cortical excitability; enhances descending inhibition via motor cortex & DLPFC	M1, DLPFC, anterior cingulate, PAG–RVM pathways	Level 2 (RCTs in chronic neuropathic pain, migraine)	Short-lived effects; high inter-individual variability; repeated sessions required	[88,91]
tDCS	Alters resting membrane potential and synaptic plasticity; strengthens inhibitory circuits	M1, DLPFC, thalamocortical networks	Level 3 (pilot studies in neuropathic pain)	Small effect size; protocol heterogeneity	[91,92]
nVNS	Stimulates cervical vagal afferents → NTS → LC & PAG → central pain/autonomic network modulation	NTS, LC, PAG, limbic circuits	Level 2 (RCTs in migraine, cluster headache)	Requires daily use; device-dependent; long-term benefit uncertain	[93,94]
TENS	Peripheral neuromodulation of large-diameter afferents; gate control & endogenous opioid activation	Cervical and trigeminal dermatomes; dorsal horn interneurons	Level 3–4 (small trials, chronic pain guidelines)	Limited efficacy in severe refractory pain; requires compliance	[23]

Abbreviations: rTMS, repetitive transcranial magnetic stimulation; tDCS, transcranial direct current stimulation; nVNS, non-invasive vagus nerve stimulation; TENS, transcutaneous electrical nerve stimulation; NTS, nucleus tractus solitarius; LC, locus coeruleus; PAG, periaqueductal gray; RVM, rostral ventromedial medulla. Level of evidence: Classified according to the Oxford Centre for Evidence-Based Medicine, where Level 1 = systematic review/RCT, Level 2 = RCT or strong observational, Level 3 = cohort/case–control, Level 4 = case series/poor-quality studies, Level 5 = expert opinion.

## Data Availability

No new data were created or analyzed in this study. Data sharing does not apply to this article.

## References

[1] IASP (2017). IASP Terminology: Neuropathic Pain Definition. International Association for the Study of Pain. https://www.iasp-pain.org/resources/terminology/.

[2] Treede R.D., Rief W., Barke A., Aziz Q., Bennett M.I., Benoliel R., Cohen M., Evers S., Finnerup N.B., First M.B. (2019). Chronic pain as a symptom or a disease: The IASP Classification of Chronic Pain for the ICD-11. Pain.

[3] Dworkin R.H., Backonja M., Rowbotham M.C., Allen R.R., Argoff C.R., Bennett G.J., Bushnell M.C., Farrar J.T., Galer B.S., Haythornthwaite J.A. (2003). Advances in neuropathic pain: Diagnosis, mechanisms, and treatment recommendations. Arch. Neurol..

[4] Jensen T.S., Baron R., Haanpää M., Kalso E., Loeser J.D., Rice A.S.C., Treede R.D. (2011). A new definition of neuropathic pain. Pain.

[5] Finnerup N.B., Attal N., Haroutounian S., McNicol E., Baron R., Dworkin R.H., Gilron I., Haanpää M., Hansson P., Jensen T.S. (2015). Pharmacotherapy for neuropathic pain in adults: A systematic review and meta-analysis. Lancet Neurol..

[6] Bouhassira D., Lantéri-Minet M., Attal N., Laurent B., Touboul C. (2008). Prevalence of chronic pain with neuropathic characteristics in the general population. Pain.

[7] van Hecke O., Austin S.K., Khan R.A., Smith B.H., Torrance N. (2014). Neuropathic pain in the general population: A systematic review of epidemiological studies. Pain.

[8] Colloca L., Ludman T., Bouhassira D., Baron R., Dickenson A.H., Yarnitsky D., Freeman R., Truini A., Attal N., Finnerup N.B. (2017). Neuropathic pain. Nat. Rev. Dis. Primers.

[9] Smith B.H., Torrance N. (2012). Epidemiology of neuropathic pain and its impact on quality of life. Curr. Pain Headache Rep..

[10] Torrance N., Smith B.H., Bennett M.I., Lee A.J. (2006). The epidemiology of chronic pain of predominantly neuropathic origin: Results from a general population survey. J. Pain.

[11] Bou Malhab F., Hosri J., Zaytoun G., Hadi U. (2025). Trigeminal cervical complex: A neural network affecting the head and neck. Eur. Ann. Otorhinolaryngol. Head Neck Dis..

[12] Bogduk N. (1992). The anatomical basis for cervicogenic headache. J. Manip. Physiol. Ther..

[13] Merskey H., Bogduk N. (1994). Classification of Chronic Pain.

[14] Piovesan E.J., Kowacs P.A., Oshinsky M.L. (2003). Convergence of cervical and trigeminal sensory afferents. Curr. Pain Headache Rep..

[15] Grgić V. (2010). Utjecaj manualne terapije vratne kraljeznice na tipicnu trigeminalnu neuralgiju: Prikaz bolesnice [Influence of manual therapy of cervical spine on typical trigeminal neuralgia: A case report]. Lijec Vjesn..

[16] Bartsch T., Goadsby P.J. (2003). The trigeminocervical complex and migraine: Current concepts and synthesis. Curr. Pain Headache Rep..

[17] Bogduk N., Govind J. (2009). Cervicogenic headache: An assessment of the evidence on clinical diagnosis, invasive tests, and treatment. Lancet Neurol..

[18] Bartsch T. (2005). Migraine and the trigeminocervical complex. Curr. Pain Headache Rep..

[19] Sessle B.J., Hu J.W., Amano N., Zhong G. (1986). Convergence of cutaneous, tooth pulp, visceral, neck and muscle afferents onto nociceptive and non-nociceptive neurones in trigeminal subnucleus caudalis (medullary dorsal horn) and its implications for referred pain. Pain.

[20] Goadsby P.J., Holland P.R., Martins-Oliveira M., Hoffmann J., Schankin C., Akerman S. (2017). Pathophysiology of Migraine: A Disorder of Sensory Processing. Physiol. Rev..

[21] Headache Classification Committee of the International Headache Society (IHS) (2018). The International Classification of Headache Disorders, 3rd edition (ICHD-3). Cephalalgia.

[22] Wei D.Y., Goadsby P.J. (2022). Recent Advances and Updates in Trigeminal Autonomic Cephalalgias. Semin. Neurol..

[23] Bendtsen L., Zakrzewska J.M., Abbott J., Braschinsky M., Di Stefano G., Donnet A., Eide P.K., Leal P.R.L., Maarbjerg S., May A. (2019). European Academy of Neurology guideline on trigeminal neuralgia. Eur. J. Neurol..

[24] Martelletti P., Katsarava Z., Lampl C., Magis D., Bendtsen L., Negro A., Russell M.B., Mitsikostas D.D., Jensen R.H. (2014). Refractory chronic migraine: A consensus statement on clinical definition from the European Headache Federation. J. Headache Pain..

[25] Ashina M., Katsarava Z., Do T.P., Buse D.C., Pozo-Rosich P., Özge A., Krymchantowski A.V., Lebedeva E.R., Ravishankar K., Yu S. (2021). Migraine: Integrated approaches to clinical management and emerging treatments. Lancet.

[26] Marciszewski K.K., Meylakh N., Di Pietro F., Macefield V.G., Macey P.M., Henderson L.A. (2017). Altered brainstem anatomy in migraine. Cephalalgia.

[27] Basedau H., Nielsen T., Asmussen K., Gloss K., Mehnert J., Jensen R.H., May A. (2022). Experimental evidence of a functional relationship within the brainstem trigeminocervical complex in humans. Pain.

[28] Kerr F.W. (1961). Structural relation of the spinal trigeminal nucleus to the upper cervical roots and the subnucleus caudalis of the nucleus of the tractus solitarius in the cat. Exp. Neurol..

[29] Dostrovsky J.O., Sessle B.J., Hu J.W. (1981). Presynaptic excitability changes produced by conditioning stimulation of primary afferent fibers in trigeminal brain-stem nuclei. J. Neurophysiol..

[30] Goadsby P.J., Hoskin K.L. (1997). The distribution of trigeminovascular afferents in the nonhuman primate brain stem. Brain.

[31] Shigenaga Y., Okamoto T., Nishimori T., Suemune S., Nasution I.D., Chen I.C., Tsuru K., Yoshida A., Tabuchi K., Hosoi M. (1986). Oral and facial representation in the trigeminal principal and rostral spinal nuclei of the cat. J. Comp. Neurol..

[32] Rhoton A.L. (2002). The supratentorial cranial space: Microsurgical anatomy and surgical approaches. Neurosurgery.

[33] DaSilva A.F., Becerra L., Makris N., Strassman A.M., Gonzalez R.G., Geatrakis N., Borsook D. (2002). Somatotopic activation in the human trigeminal pain pathway. J. Neurosci..

[34] Fernandes E.C., Carlos-Ferreira J., Luz L.L., Kokai E., Meszar Z., Szucs P., Safronov B.V. (2022). Processing of trigeminocervical nociceptive afferent input by neuronal circuity in the upper cervical lamina I. Pain.

[35] Russo A.F. (2015). Calcitonin gene-related peptide (CGRP): A new target for migraine. Annu. Rev. Pharmacol. Toxicol..

[36] Edvinsson L. (2019). CGRP antibodies as prophylaxis in migraine. Lancet Neurol..

[37] Oshinsky M.L., Gomonchareonsiri S. (2007). Episodic dural stimulation in awake rats: A model for recurrent headache. Headache.

[38] May A., Goadsby P.J. (2002). The trigeminovascular system in humans: Pathophysiologic implications for primary headache syndromes of the neural influences on the cerebral circulation. J. Cereb. Blood Flow. Metab..

[39] Woolf C.J. (2011). Central sensitization: Implications for the diagnosis and treatment of pain. Pain.

[40] Bliss T.V., Collingridge G.L., Kaang B.K., Zhuo M. (2016). Synaptic plasticity in the anterior cingulate cortex in acute and chronic pain. Nat. Rev. Neurosci..

[41] Gao Y.J., Ji R.R. (2010). Chemokines, neuronal–glial interactions, and central processing of neuropathic pain. Pharmacol. Ther..

[42] Milligan E.D., Watkins L.R. (2009). Pathological and protective roles of glia in chronic pain. Nat. Rev. Neurosci..

[43] Devor M. (2006). Sodium channels and mechanisms of neuropathic pain. J. Pain.

[44] Ossipov M.H., Dussor G.O., Porreca F. (2010). Central modulation of pain. J. Clin. Investig..

[45] Borsook D., Maleki N., Becerra L., McEwen B. (2013). Understanding migraine through the lens of maladaptive stress responses: A model disease of allostatic load. Neuron.

[46] Xiong N.X., Zhao H.Y., Zhang F.C., Liu R.E. (2012). Vagoglossopharyngeal neuralgia treated by microvascular decompression and glossopharyngeal rhizotomy: Clinical results of 21 cases. Stereotact. Funct. Neurosurg..

[47] Woda A., Pionchon P. (1999). A unified concept of idiopathic orofacial pain: Clinical features. J. Orofac. Pain.

[48] Okeson J.P. (2005). Bell’s Orofacial Pains: The Clinical Management of Orofacial Pain.

[49] Travell J.G., Simons D.G. (1992). Myofascial Pain and Dysfunction: The Trigger Point Manual.

[50] Jääskeläinen S.K. (2021). Clinical neurophysiology and quantitative sensory testing in the investigation of orofacial pain and sensory function. J. Oral Rehabil..

[51] Taziki M.H., Behnampour N. (2012). A study of the etiology of referred otalgia. Iran. J. Otorhinolaryngol..

[52] Biondi D.M. (2005). Cervicogenic headache: A review of diagnostic and treatment strategies. J. Am. Osteopath. Assoc..

[53] Choi H.J., Oh I.H., Choi S.K., Lim Y.J. (2012). Clinical outcomes of pulsed radiofrequency neuromodulation for the treatment of occipital neuralgia. J. Korean Neurosurg. Soc..

[54] Slavin K.V., Isagulyan E.D., Gomez C., Yin D. (2019). Occipital Nerve Stimulation. Neurosurg. Clin. N. Am..

[55] Maixner W., Fillingim R.B., Williams D.A., Smith S.B., Slade G.D. (2016). Overlapping Chronic Pain Conditions: Implications for Diagnosis and Classification. J. Pain.

[56] Renton T. (2011). Dental (Odontogenic) Pain. Rev. Pain.

[57] Daly B.J., Sharif M.O., Jones K., Worthington H.V., Beattie A. (2022). Local interventions for the management of alveolar osteitis (dry socket). Cochrane Database Syst. Rev..

[58] Melis M., Lobo S.L., Ceneviz C., Zawawi K., Al-Badawi E., Maloney G., Mehta N. (2003). Atypical odontalgia: A review of the literature. Headache.

[59] Schreiber C.P., Hutchinson S., Webster C.J., Ames M., Richardson M.S., Powers C. (2004). Prevalence of migraine in patients with a history of self-reported or physician-diagnosed “sinus” headache. Arch. Intern. Med..

[60] Fokkens W.J., Lund V.J., Hopkins C., Hellings P.W., Kern R., Reitsma S., Toppila-Salmi S., Bernal-Sprekelsen M., Mullol J., Alobid I. (2020). European Position Paper on Rhinosinusitis and Nasal Polyps 2020. Rhinology.

[61] Rushton J.G., Stevens J.C., Miller R.H. (1981). Glossopharyngeal (vagoglossopharyngeal) neuralgia: A study of 217 cases. Arch. Neurol..

[62] Wang X., Meng D., Wang L., Chen G. (2021). The Clinical Characteristics and Surgical Treatment of Glossopharyngeal Neuralgia With Pain Radiating to the Innervated Area of the Trigeminal Nerve. J. Oral. Maxillofac. Surg..

[63] Eagle W.W. (1937). Elongated styloid process: Report of two cases. Arch. Otolaryngol..

[64] Badhey A., Jategaonkar A., Anglin Kovacs A.J., Kadakia S., De Deyn P.P., Ducic Y., Schantz S., Shin E. (2017). Eagle syndrome: A comprehensive review. Clin. Neurol. Neurosurg..

[65] Watson C.P. (1998). Postherpetic neuralgia: The importance of preventing this intractable end-stage disorder. J. Infect. Dis..

[66] Saguil A., Kane S., Mercado M., Lauters R. (2017). Herpes Zoster and Postherpetic Neuralgia: Prevention and Management. Am. Fam. Physician.

[67] Ashina M., Katsarava Z., Do T.P., Buse D.C., Pozo-Rosich P., Özge A., Krymchantowski A.V., Lebedeva E.R., Ravishankar K., Yu S. (2021). Migraine: Epidemiology and systems of care. Lancet.

[68] Yanagisawa K., Kveton J.F. (1992). Referred otalgia. Am. J. Otolaryngol..

[69] Ramazani F., Szalay-Anderson C., Batista A.V., Park P., Hwang E., Chau J., Lui J. (2023). Referred otalgia: Common causes and evidence-based strategies for assessment and management. Can. Fam. Physician.

[70] Travell J.G., Simons D.G. (1999). Myofascial Pain and Dysfunction: The Trigger Point Manual.

[71] Giamberardino M.A., Affaitati G., Fabrizio A., Costantini R. (2011). Myofascial pain syndromes and their evaluation. Best. Pract. Res. Clin. Rheumatol..

[72] Amer T.A., El-Shmam O.M. (1997). Chiari malformation type I: A new MRI classification. Magn. Reson. Imaging.

[73] Hwang L., Dessouky R., Xi Y., Amirlak B., Chhabra A. (2017). MR Neurography of Greater Occipital Nerve Neuropathy: Initial Experience in Patients with Migraine. AJNR Am. J. Neuroradiol..

[74] Naja Z.M., El-Rajab M., Al-Tannir M.A., Ziade F.M., Tawfik O.M. (2006). Occipital nerve blockade for cervicogenic headache: A double-blind randomized controlled clinical trial. Pain Pract..

[75] Vanelderen P., Rouwette T., De Vooght P., Puylaert M., Heylen R., Vissers K., Van Zundert J. (2010). Pulsed radiofrequency for the treatment of occipital neuralgia: A prospective study with 6 months of follow-up. Reg. Anesth. Pain Med..

[76] Kosinski M., Bayliss M.S., Bjorner J.B., Ware J.E., Garber W.H., Batenhorst A., Cady R., Dahlöf C.G., Dowson A., Tepper S. (2003). A six-item short-form survey for measuring headache impact: The HIT-6. Qual. Life Res..

[77] Mustafa M.S., Bin Amin S., Kumar A., Shafique M.A., Fatima Zaidi S.M., Arsal S.A., Rangwala B.S., Iqbal M.F., Raja A., Haseeb A. (2024). Assessing the effectiveness of greater occipital nerve block in chronic migraine: A systematic review and meta-analysis. BMC Neurol..

[78] Schwedt T.J., Dodick D.W., Hentz J., Trentman T.L., Zimmerman R.S. (2007). Occipital nerve stimulation for chronic headache—Long-term safety and efficacy. Cephalalgia.

[79] Fontaine D., Sol J.C., Raoul S., Fabre N., Geraud G., Magne C., Sakarovitch C., Lanteri-Minet M. (2011). Treatment of refractory chronic cluster headache by chronic occipital nerve stimulation. Cephalalgia.

[80] Deer T.R., Mekhail N., Provenzano D., Pope J., Krames E., Leong M., Levy R.M., Abejon D., Buchser E., Burton A. (2014). The appropriate use of neurostimulation of the spinal cord and peripheral nervous system for the treatment of chronic pain and ischemic diseases: The Neuromodulation Appropriateness Consensus Committee. Neuromodulation.

[81] Mekhail N., Levy R.M., Deer T.R., Kapural L., Li S., Amirdelfan K., Hunter C.W., Rosen S.M., Costandi S.J., Falowski S.M. (2020). Long-term safety and efficacy of closed-loop spinal cord stimulation to treat chronic back and leg pain (Evoke): A double-blind, randomised, controlled trial. Lancet Neurol..

[82] Cruccu G., Sommer C., Anand P., Attal N., Baron R., Garcia-Larrea L., Haanpaa M., Jensen T.S., Serra J., Treede R.D. (2010). EFNS guidelines on neuropathic pain assessment: Revised 2009. Eur. J. Neurol..

[83] Lefaucheur J.P., Drouot X., Cunin P., Bruckert R., Lepetit H., Créange A., Wolkenstein P., Maison P., Keravel Y., Nguyen J.P. (2009). Motor cortex stimulation for the treatment of refractory peripheral neuropathic pain. Brain.

[84] Rasche D., Tronnier V.M. (2016). Clinical Significance of Invasive Motor Cortex Stimulation for Trigeminal Facial Neuropathic Pain Syndromes. Neurosurgery.

[85] Rasche D., Ruppolt M., Stippich C., Unterberg A., Tronnier V.M. (2006). Motor cortex stimulation for long-term relief of chronic neuropathic pain: A 10 year experience. Pain.

[86] Parmar V.K., Gee L., Smith H., Pilitsis J.G. (2014). Supraspinal stimulation for treatment of refractory pain. Clin. Neurol. Neurosurg..

[87] Boccard S.G., Fitzgerald J.J., Pereira E.A., Moir L., Van Hartevelt T.J., Kringelbach M.L., Green A.L., Aziz T.Z. (2014). Targeting the affective component of chronic pain: A case series of deep brain stimulation of the anterior cingulate cortex. Neurosurgery.

[88] Lefaucheur J.P., Aleman A., Baeken C., Benninger D.H., Brunelin J., Di Lazzaro V., Filipović S.R., Grefkes C., Hasan A., Hummel F.C. (2020). Evidence-based guidelines on the therapeutic use of repetitive transcranial magnetic stimulation (rTMS): An update (2014–2018). Clin. Neurophysiol..

[89] Halker Singh R.B., Ailani J., Robbins M.S. (2019). Neuromodulation for the Acute and Preventive Therapy of Migraine and Cluster Headache. Headache.

[90] Gatchel R.J., Peng Y.B., Peters M.L., Fuchs P.N., Turk D.C. (2007). The biopsychosocial approach to chronic pain: Scientific advances and future directions. Psychol. Bull..

[91] Fregni F., Pascual-Leone A. (2007). Technology insight: Noninvasive brain stimulation in neurology—Perspectives on the therapeutic potential of rTMS and tDCS. Nat. Clin. Pract. Neurol..

[92] Garcia-Larrea L., Peyron R. (2007). Motor cortex stimulation for neuropathic pain: From phenomenology to mechanisms. Neuroimage.

[93] Silberstein S.D., Mechtler L.L., Kudrow D.B., Calhoun A.H., McClure C., Saper J.R., Liebler E.J., Rubenstein Engel E., Tepper S.J., ACT1 Study Group (2016). Non-Invasive Vagus Nerve Stimulation for the Acute Treatment of Cluster Headache: Findings From the Randomized, Double-Blind, Sham-Controlled ACT1 Study. Headache.

[94] Najib U., Smith T., Hindiyeh N., Saper J., Nye B., Ashina S., McClure C.K., Marmura M.J., Chase S., Liebler E. (2022). Non-invasive vagus nerve stimulation for prevention of migraine: The multicenter, randomized, double-blind, sham-controlled PREMIUM II trial. Cephalalgia.

[95] Borsook D., Erpelding N., Becerra L. (2013). Losses and gains: Chronic pain and altered brain morphology. Expert. Rev. Neurother..

[96] Tansley S., Uttam S., Ureña Guzmán A., Yaqubi M., Pacis A., Parisien M., Deamond H., Wong C., Rabau O., Brown N. (2022). Single-cell RNA sequencing reveals time- and sex-specific responses of mouse spinal cord microglia to peripheral nerve injury and links ApoE to chronic pain. Nat. Commun..

[97] Fiore N.T., Yin Z., Guneykaya D., Gauthier C.D., Hayes J.P., D’Hary A., Butovsky O., Moalem-Taylor G. (2022). Sex-specific transcriptome of spinal microglia in neuropathic pain due to peripheral nerve injury. Glia.

[98] Saitoh Y., Hirayama A., Kato A., Kishima H., Shimokawa T., Oshino S., Hirata M., Tani N., Kato A., Yoshimine T. (2007). Motor cortex stimulation for central and peripheral deafferentation pain. Pain.

[99] Velasco F., Velasco M., Jiménez F., Carrillo-Ruiz J.D., Castro G., Velasco A.L. (2008). Efficacy of motor cortex stimulation in the treatment of neuropathic pain: A randomized double-blind trial. J. Neurosurg..

[100] Johnson M.D., Burchiel K.J. (2014). Peripheral nerve stimulation for treatment of occipital neuralgia and other chronic headaches. Neuromodulation.

[101] Slotty P.J., Bara G., Walter J., Vesper J. (2015). Stimulation of the auriculotemporal nerve in patients with refractory cluster headache. Cephalalgia.

[102] Popiolek-Barczyk K., Mika J. (2016). Targeting the Microglial Signaling Pathways: New Insights in the Modulation of Neuropathic Pain. Curr. Med. Chem..

[103] Kumbhare D.A., Elzibak A.H., Noseworthy M.D. (2017). Evaluation of Chronic Pain Using Magnetic Resonance (MR) Neuroimaging Approaches: What the Clinician Needs to Know. Clin. J. Pain.

